# Prognostic impact of MYD88 and TP53 mutations in diffuse large B Cell lymphoma

**DOI:** 10.1007/s00277-023-05420-1

**Published:** 2023-09-02

**Authors:** Osama Abd El Hameed Ebid, Lobna R. Ezz El Arab, Amr S. Saad, Mai Ezz El Din, Nermeen Mostafa, Menha Swellam

**Affiliations:** 1 Clinical Oncology Department, Faculty of Medicine, Ain Shams University, Cairo, Egypt; 2 Biochemistry Department, Biotechnology Research Institute, High Throughput Molecular and Genetic Technology Laboratory, Central Laboratories Network and the Centers of Excellence, National Research Centre, Dokki, Giza, 12622 Egypt

**Keywords:** MYD88, TP53, DLBCL, Prognosis, Response

## Abstract

Diffuse large B cell lymphoma (DLBCL) is the most common subtype of lymphoma. It is a highly heterogeneous lymphoid neoplasm, with variations in gene expression profiles and genetic alterations. MYD88 and TP53 genes are common to be expressed and mutated in DLBCL patients with controversy regarding their role in prognosis and survival. This study aims to determine the predictive and prognostic role of MYD88 and TP53 gene mutation in DLBCL. A prospective cohort study was conducted on 50 patients who were diagnosed with DLBCL and 30 healthy individuals to assess the sensitivity and specificity of MYD88 and TP53 genetic mutations. MYD88 and TP53 gene mutations were more sensitive, specific, and accurate in predicting overall mortality and disease progression in comparison with the international prognostic index. Mutant MYD88 and TP53 showed their prognostic importance for worse objective response rates and survival outcomes. Both mutant MYD88 and TP53 were associated with worse ORR. There was a significant statistical difference for both MYD88 and TP53 with regard to 2-year PFS and 2-year OS rate. Hence, both mutant MYD88 and TP53 can be used in predicting disease progression and overall mortality.

## Introduction

Non-Hodgkin lymphoma (NHL) is the commonest hematologic malignancy in the world. It is the third most common malignancy in Egypt. According to Globocan 2020, the estimated number of new cases is 7305 cases, while the estimated rate of deaths in 2020 is 89,042. The most common subtype of lymphoma in adults worldwide is diffuse large B cell lymphoma, composing about one-third of NHL, diagnosed each year [1].

Diffuse large B cell lymphoma can be subdivided by gene expression profile (GEP) or different immunohistochemistry (IHC) algorithms into germinal central B cell (GCB) like and activated B cell (ABC) like subtypes, with the ABC having a significantly worse prognosis than the GCB group [2]. The revised World Health Organization (WHO) classification of tumors of hematopoietic and lymphoid tissue now includes a requirement for the characterization of molecular features of clinical significance, such as the cell of origin and the rearrangements of the MYC, BCL2, and BCL6 genes. Schmitz and colleagues [3] identified four distinctive genetic subtypes of disease with different recurring mutations portending to differential clinical outcomes by performing WES, array-based DNA copy number analysis, and targeted amplicon re-sequencing on 574 primarily pre-treatment DLBCL biopsy samples. Wright et al. (2020) [4] developed the Lymph-Gen algorithm to divide DLBCL into seven genetic subtypes including MCD, N1, A53, BN2 (BCL6 translocations and NOTCH2 mutations), ST2, and EZB-MYC. The MCD subtype was characterized by the co-occurrence of myeloid differentiation primary response 88 (MYD88) (L265P) and CD79 mutations. Patients with MCD subtype had a poor prognosis [5]. Myeloid differentiation primary response 88-L265P mutations are more frequent in DLBCLs located at specific extra-nodal sites, including in the central nervous system [6], breast [7], skin [8], and testis [9]. Different studies have demonstrated that MYD88 mutations are associated with inferior OS in DLBCLs patients in comparison to wild-type MYD88 [10]. Other studies have shown the utility of MYD88 L265P in prognosis and response to therapy [11]. Xu et al. (2017) demonstrated that MYD88 mutations were significantly more common in DLBCL patients who were refractory to chemotherapy with R-CHOP (rituximab, cyclophosphamide, doxorubicin, vincristine, and prednisone) compared with DLBCL patients who were chemo-sensitive (15%). However, another study [12] showed that MYD88 L265P mutation had a favorable effect. Therefore, the prognostic effect of MYD88 L265P mutation warrants further study [13]. The tumor suppressor P53 (TP53) is also known as the guardian of the genome. It acts as a sensor of cellular homeostasis and plays important roles in DNA repair, senescence, metabolism, and induction of apoptotic and non-apoptotic cell death in order to maintain the integrity of the genome and normal cellular functions [14]. TP53 mutations occur in 20 to 25% of DLBCL patients and have been identified as one of the most commonly mutated genes in both GCB and ABC subtypes in DLBCL patients [15]. TP53 mutation has been identified as an unfavorable prognostic factor for patients receiving CHOP or R-CHOP treatment regimens [16]. The prognostic significance of TP53 mutations was further confirmed in the RICOVER 60 trial, suggesting the use of TP53 mutations for prognostication and treatment stratification [16]. However another study does not confirm these findings [17].

 In order to address this issue, this study was conducted with the following aims: detect the presence of MYD88 and TP53 gene mutations and correlate these findings with clinical outcome, initial treatment response, overall survival, and progression-free survival in a cohort of 50 DLBCL patients.

## Patients and method

This was a prospective cohort study that included 80 individuals who were divided into 50 adult cases of DLBCL attending at the Department of Clinical Oncology Ain Shams University Hospitals and 30 healthy controls who were free from any malignancy and matched with cases by age. The study was approved by the ethical committee of Ain Shams University (FWA 000017585). The inclusion criteria were patients diagnosed with ECOG 0–2, age >18 years, < 65 years, adequate liver functions, negative viral markers (hepatitis B virus surface antigen, hepatitis C virus antibody), and with normal baseline Echo, while exclusion criteria were those patients who had double primary malignancy, pregnant female patients, and those who refused to sign informed consent or contraindication for RCHOP.

### Sample collection

After obtaining approval from the ethical medical committee, all enrolled individuals (50 DLBCL patients and 30 healthy individuals) agreed to participate in the study, and informed written consent was obtained from each individual for the use of their blood specimens, Peripheral blood (3 ml) was collected from DLBCL patients in vacutainer blood collection tubes containing EDTA and transported to the laboratory within 30 min in ice containers. After centrifugation of blood samples at 10,000×g for 10 min at 4°C (3-18KS, Sigma, Germany) the plasma (200 μL) was separated and stored in aliquots and stored immediately at −80°C until further processing.

### DNA extraction

DNA extraction was carried out using QIAamp DNA Mini kit (cat no. 51104, Qiagen, Hilden, Germany), according to manufacturer guidelines based on the spin column technique for DNA extraction. The total DNA concentration was detected using a spectrophotometer Nano-drop (Quawell, Q-500, Scribner, USA) as (1.03–14.6 ng/μL) and stored at −20 °C until further assessments.

### Assessment of MYD88 mutation

Mutation of MYD88 was amplified by using TaqDNA polymerase (Thermo-fisher scientific-company) using primers for MYD88: Forward (F): 5\-CTGGCAAGAGAATGAGGGAATGT-3\ and Reverse (R) 5\ AGGAGGCAGGGCAGAAGTA-3\. Thermal profile conditions were done with some modifications as follows: initial denaturation at 95°C for 10 min (one cycle) then 35 cycles of 95°C for 30 s, 56°C for 30 s, and final elongation at 72°C for 1 min. The digestion using restriction enzyme BsiEI (Thermo Scientific Fast Digest BSH1285I, cat no. FD0894) was added to the reaction mixture. Two methods were used for the detection of MYD88 mutation. The first method was using the digested PCR products were run on agarose gel 2% and detected on the gel documentation system (Syngene, G:BOX F3, USA). Interpretation for the results depends on the amplification of exon 5 of the MYD88 gene that produces a 489-bp product, so it is seen in all cases. But the presence of MYD88 L265P mutation results in the development of BsiEI restriction enzyme site; thus, the mutated allele results in the creation of 289-bp and 200-bp extra fragments. The 2nd method amplified the digested product then the melting curve of the digested versus the undigested was compared.

### Detection of TP53 mutation using high-resolution melting

Mutation of TP53 was detected using the high-resolution melting (HRM) method which consists of performing the PCR in the existence of DNA binding dye LC Green®, observing the progressive alteration in the fluorescence produced by delivery of the dye from a DNA duplex as it is denatured by increasing the temperature, collecting HRM curve, and recognizing the samples with melting curve aberrations revealing the occurrence of a sequence variant. Fluorescence intensity as a function of temperature, examined by the LightScanner® instrument (Idaho Technology, Salt Lake City, UT, USA), can expose very small variations in the melting curve shape when investigated with the LightScanner® software using the “scanning” mode (Idaho Technology, Salt Lake City, UT, USA). Accordingly, HRM reactions of total volume 20 μL were carried out with 18 ng of extracted DNA, 7.47pmol/L of each P53 primer (TP53 F 5\-CTGTGGGAAGCGAAAATTCCATGG-3\, R5\ACTTCCTGAAAACAACGTTCTGG-3\), and 10 μL of MeltDoctorTM HRM master mix (Applied Biosystems, USA, cat no. 4415440) according to the manufacturer protocol.

### Patient cohort and treatment strategy

**Figure 1:**
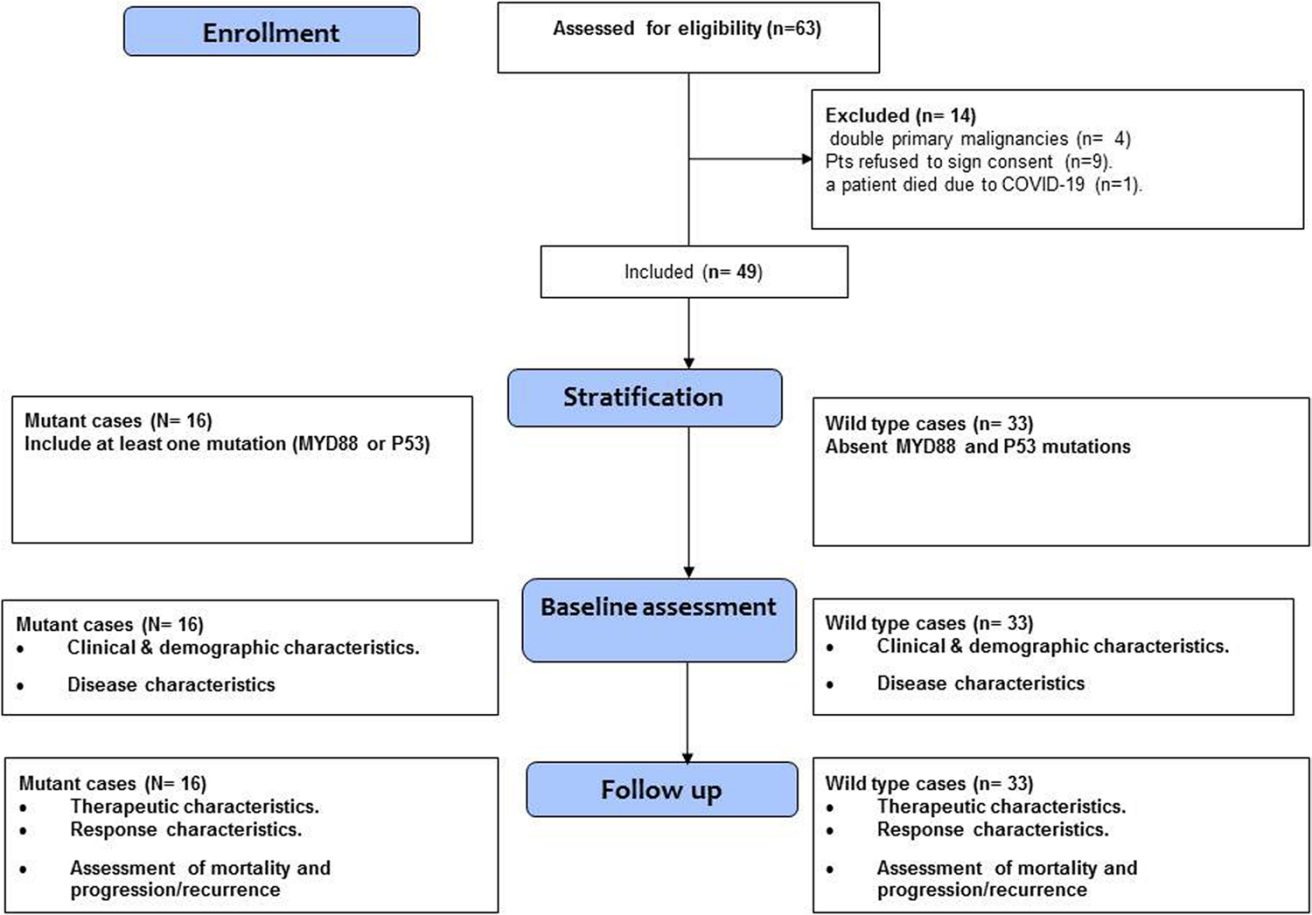
Study flow chart

At baseline, the demographic, clinical, and pathological data were collected. All patients were staged according to Ann Arbor staging system, with baseline PET/CT scan. Treatment protocols were as follows: stage I–II received 3–6 cycles of chemo-immunotherapy with or without involved site radiotherapy to the initial bulky sites, and stages III–IV received 6 cycles of chemo-immunotherapy with involved site radiotherapy to initial bulky sites and to sites of extra-nodal involvement. The chemo-immunotherapy protocol used was RCHOP, with or without CNS prophylaxis. The median follow-up time for the cases was estimated at 24 months, and responses were defined by Lugano classification response criteria [18]. Patients were followed up every 3 months and their outcomes (response, PFS, OS) were recorded and included in the analysis, as shown in Fig. 1.

### Statistical analysis

Descriptive statistics was carried out as the continuous data was described using the mean (SD) and median interquartile range (IQR). Analytical statistics was assessed using chi-square or Fischer exact tests to compare nominal data. Wilcoxon test was implemented for comparing continuous variables. For subgroup analysis of different patterns of mutations, Fischer exact test, Kruskal Wallis, and ANOVA were utilized for hypothesis testing. The *P* value was considered significant at 0.05. Kaplan-Meier method was used to estimate the progression-free survival and overall survival across wild and mutant groups using log-rank tests. Logistic regression models were applied to determine the predictive performance of MYD88 and TP53 with or without IPI for both death and disease progression. Comparison between the candidate logistic models was based on the area under the curve (AUC) of the receiver operating characteristic curve (ROC), sensitivity, specificity, and accuracy. All analyses were performed using R (Version 4.1.4, Foundation for Statistical Computing, Vienna, Austria) and SPSS for Windows (Version 26, IBM, NY, USA).

## Results

The study was conducted on two groups, the first group was DLBCL patients (*n*=49), and the mean age of the studied patients was 51.04 ± 12.39 years, of them 21 patients (42.86%) were males. Seven patients (14.29%) reported having a family history. Co-morbidities were present in 23 patients (46.94%) of them 17 patients were hypertensive, and 6 patients were diabetic, summary for enrolled DLBCL patients is shown in (Table 1), The second group was healthy control individuals (*n*=30), with a mean age 45 ± 10.5, of them 66.67% males. In DLBCL patients, the prevalence of MYD88 mutations was 18.37%, while the prevalence of TP53 mutations was 28.57%), as shown in Table 1, and in the healthy control group, both MYD88 and TP53 were negative for mutations (100% wild type). Mutations versus wild-type patterns of TP53 and MYD88 genes were categorized as follows: wild for both MYD88 and TP53 (WW; 33 patients [67.3%]), mutant for both TP53 and MYD88 genes (MM; 7 patients [14.3%]), TP53 mutant and MYD88 wild cases (7 patients [14.3%]), and TP53 wild and MYD88 mutant cases (2 cases [4.1%]). There were no statistically significant differences regarding age, sex, family history, and comorbidities among these different patterns of mutations (Table 2).

**Table 1:** Baseline demographic and clinical characteristics of the study group (DLBCL *N*=49) (healthy *N*=30)

Characteristic	*N* = 49^1^
Age (years)	
*N*	49.00
Median (IQR)	51 (43, 60)
Range	20, 76
Mean (SD)	51.04 (12.39)
Gender	
Female	28 (57.14%)
Male	21 (42.86%)
Family history	7 (14.29%)
Comorbidity	23 (46.94%)
MYD88	
Wild	40 (81.63%)
Mutant	9 (18.37%)
TP53	
Wild	35 (71.43%)
Mutant	14 (28.57%)
Genotype	
Wild	33 (67.35%)
Mutant	16 (32.65%)

^1^*n* (%)

**Table 2:** Comparing some of the baseline demographic characteristics among different patterns of mutations

Characteristic	WW	MM	MYD88 mutant	TP53 mutant	*P* value*
Age					0.7
*N*	33	7	2	7	
Median (IQR)	51.00 (43–59)	47.00 (45.5–64.50)	58.50 (54.75–62.25)	45.00 (37.5–55.50)	
Range	20–76	30–66	51–66	30–67	
Mean (SD)	51.3 (12.32)	51.86 (13.58)	58.5 (10.61)	46.86 (13.18)	
Gender					0.8
Female	19 (57.58%)	3 (42.86%)	1 (50%)	5 (71.43%)	
Male	14 (42.42%)	4 (57.14%)	1 (50%)	2 (28.57%)	
Family history	3 (9.09%)	2 (28.57%)	1 (50%)	1 (14.29%)	0.2
Comorbidity	13 (39.39%)	5 (71.43%)	0 (0%)	5 (71.43%)	0.11

Statistical analysis was calculated using the Kruskal-Wallis rank sum test and Fisher’s exact test

*WW,* wild type, *MM*, mutant type

Most of the wild patients were presented with an ECOG score of 1 (84.9%), while 62.5% of mutant patients were presented with a score of 2 (*P*<0.001). About 66.7% of the wild cases were presented with a cancer stages I–II, while 68.8% of mutant cases were presented with a cancer stages III–IV (*P*=0.019). Therefore, the IPI scores were significantly higher in mutant vs. wild cases (*P*=0.028). There were no significant differences between wild vs. mutant cases regarding the incidence of extra-nodal involvement (*P*=0.13) and LDH abnormality (*P*=0.13). By comparing the mutation among the two genes and prognostic indicators (Table 3), the MM group (i.e., mutant in MYD88 and TP53) was presented with the worst prognosis in terms of ECOG score (*P*<0.001) and cancer stage (*P*=0.04).

**Table 3:** Comparing prognostic indicators between different patterns of mutations (*N*=49)

Characteristic	WW (*n*=33)	MM (*n*=7)	MYD88 mutant (*n*=2)	TP53 mutant (*n*=7)	*P* value^2^
ECOG					**<0.001**
1	28 (84.85%)	2 (28.57%)	1 (50.00%)	2 (28.57%)	
2	5 (15.15%)	5 (71.43%)	1 (50.00%)	5 (71.43%)	
Cancer stage					**0.04**
I	10 (30.30%)	0 (0%)	0 (0%)	0 (0%)	
IE	7 (21.21%)	1 (14.29%)	0 (0%)	1 (14.29%)	
II	5 (15.15%)	1 (14.29%)	0 (0%)	0 (0%)	
IIE	0 (0.00%)	1 (14.29%)	0 (0%)	1 (14.29%)	
III	5 (15.15%)	1 (14.29%)	1 (50.00%)	3 (42.86%)	
IIIE	0 (0%)	0 (0%)	1 (50.00%)	0 (0%)	
IV	4 (12.12%)	1 (14.29%)	0 (0%)	1 (14.29%)	
IVE	2 (6.06%)	2 (28.57%)	0 (0%)	1 (14.29%)	
Stage category					0.076
1–2	22 (66.67%)	3 (42.86%)	0 (0%)	2 (28.57%)	
3–4	11 (33.33%)	4 (57.14%)	2 (100.00%)	5 (71.43%)	
Extra-nodal involvement					0.4
Positive	13 (39.39%)	5 (71.43%)	1 (50.00%)	4 (57.14%)	
Negative	20 (60.61%)	2 (28.57%)	1 (50.00%)	3 (42.86%)	
LDH					0.11
High	13 (39.39%)	5 (71.43%)	0 (0%)	5 (71.43%)	
Normal	20 (60.61%)	2 (28.57%)	2 (100%)	2 (28.57%)	
IPI					0.12
0	8 (24.24%)	0 (0%)	0 (0%)	0 (0%)	
1–2	23 (69.70%)	4 (57.14%)	2 (100%)	6 (85.71%)	
3–5	2 (6.06%)	3 (42.86%)	0 (0%)	1 (14.29%)	

Statistical analysis was calculated using the Kruskal-Wallis rank sum test and Fisher’s exact test

*WW*, wild type, *MM*, mutant type

**Figure 2:**
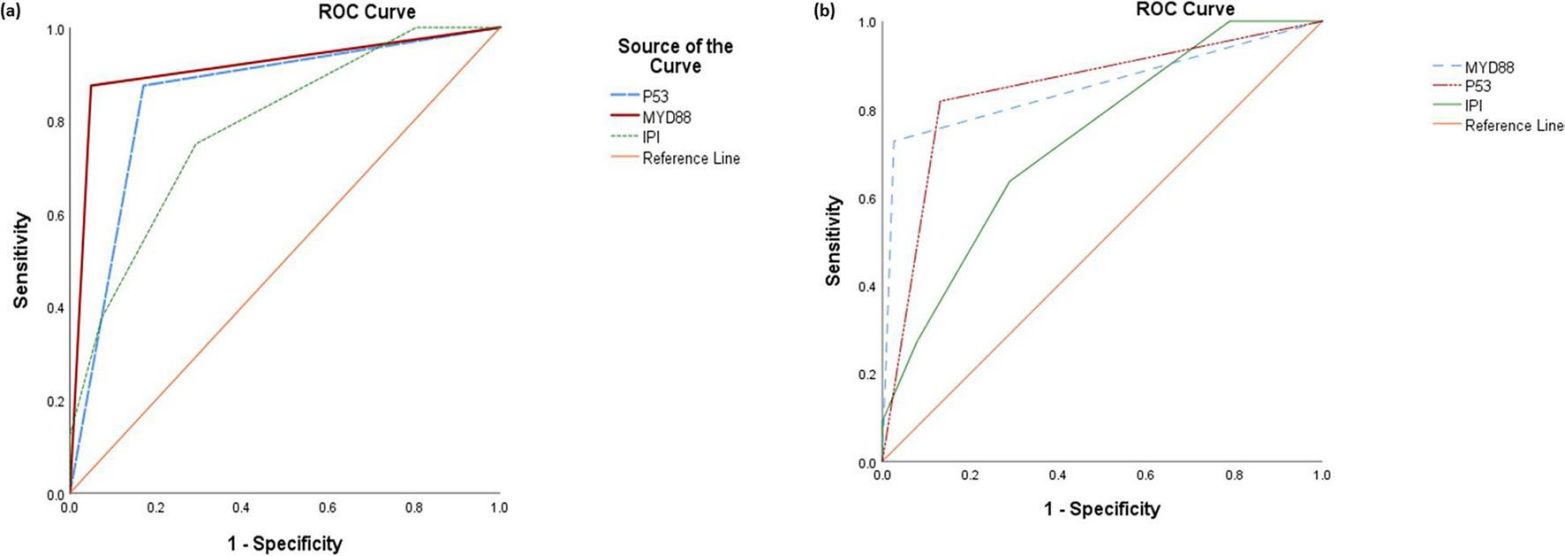
ROC curves comparing the predictive capacity of gene mutations and IPI for **a** overall mortality and **b** progression/recurrence

Regarding prognostic accuracy of gene mutations, the highest sensitivity, specificity, and prognostic accuracy for predicting the overall mortality were associated with MYD88 mutations as evidenced by the highest area under receiver operating curves (AUC ROC=0.91, *P*<0.001, 95.1%, 87.5%, 93.9% respectively). The lowest sensitivity, specificity, and prognostic accuracy were associated with IPI as evidenced by the lowest area under receiver operating curves (AUCROC=0.79, *P*= 0.011, 70.7%, 75%, 71.4% respectively). TP53 mutations demonstrated intermediate sensitivity, specificity, and prognostic accuracy between MYD88 and IPI as evidenced from the intermediate area under the receiver operating curve (AUC ROC=0.85, *P*= 0.002, 82.9%, 87.5%, 83.7% respectively). Similarly, the highest sensitivity and prognostic accuracy for predicting the progression were associated with MYD88 mutations (AUC ROC=0.85, *P*<0.001, 92.5%, 90.7% respectively). The lowest sensitivity and prognostic accuracy were associated with IPI (AUC ROC=0.73, *P*= 0.021, 79.2%, 79.6% respectively). TP53 mutations demonstrated intermediate sensitivity and prognostic accuracy among MYD88 and IPI (AUC ROC=0.84, *P*=0.001, 94.3%, 85.7% respectively) (Table 4, Fig. 2a and b). In order to allow for IPI incorporation into logistic regression models, IPI scores were dichotomized into two categories: reference category (IPI 0–2) and second category (IPI 3–5).

**Table 4:** Comparing the prognostic accuracy of different gene mutations vs. IPI in predicting overall mortality/ progression/recurrence

Marker	AUCROC Overall mortality	*P*	Sensitivity	Specificity	Accuracy	AUCROC progression/recurrence	*P*	Sensitivity	Specificity	Accuracy
MYD88	0.91	**<0.001**	95.1%	87.5%	93.9%	0.85	**<0.001**	92.5%	88.9%	90.7%
TP53	0.85	**0.002**	82.9%	87.5%	83.7%	0.84	**0.001**	94.3%	64.3%	85.7%
IPI	0.79	**0.011**	70.7%	75%	71.4%	0.73	**0.021**	79.2%	100%	79.6%

*IPI*, international prognostic index

When comparing the response characteristics across the different patterns of mutations, MM cases were presented with the lowest rate of complete response (28.6%), the highest rate of progressive disease (42.7%), and the shortest OS (28.6%). On the contrary, WW was presented with the highest rate of complete response (81.8%), the highest rate of partial response (12.1%), the lowest rates of progressive disease (0%), and the shortest OS (3.03%) (*P*=0.002). There were statistically significant differences among the different patterns in terms of overall mortality (*P*<0.001) and disease progression (*P*<0.001). The highest rates of disease progression and mortality were presented with MM cases (85.7% and 100%, respectively). Alternatively, the lowest rates of disease progression and mortality were presented with WW cases (0%, 3.03%, respectively).

The 2-year OS survival rate for the studied patients was 88% (95% CI: 79–99%), while the 2-year progression-free survival was 86% (95% CI: 77–97%). There was a significantly lower 2-year survival in MYD88 mutant vs. wild cases (33% vs. 100%, *P*<0.001) and in TP53mutant vs. wild cases (70%vs. 96%., *P*<0.001).

**Figure 3:**
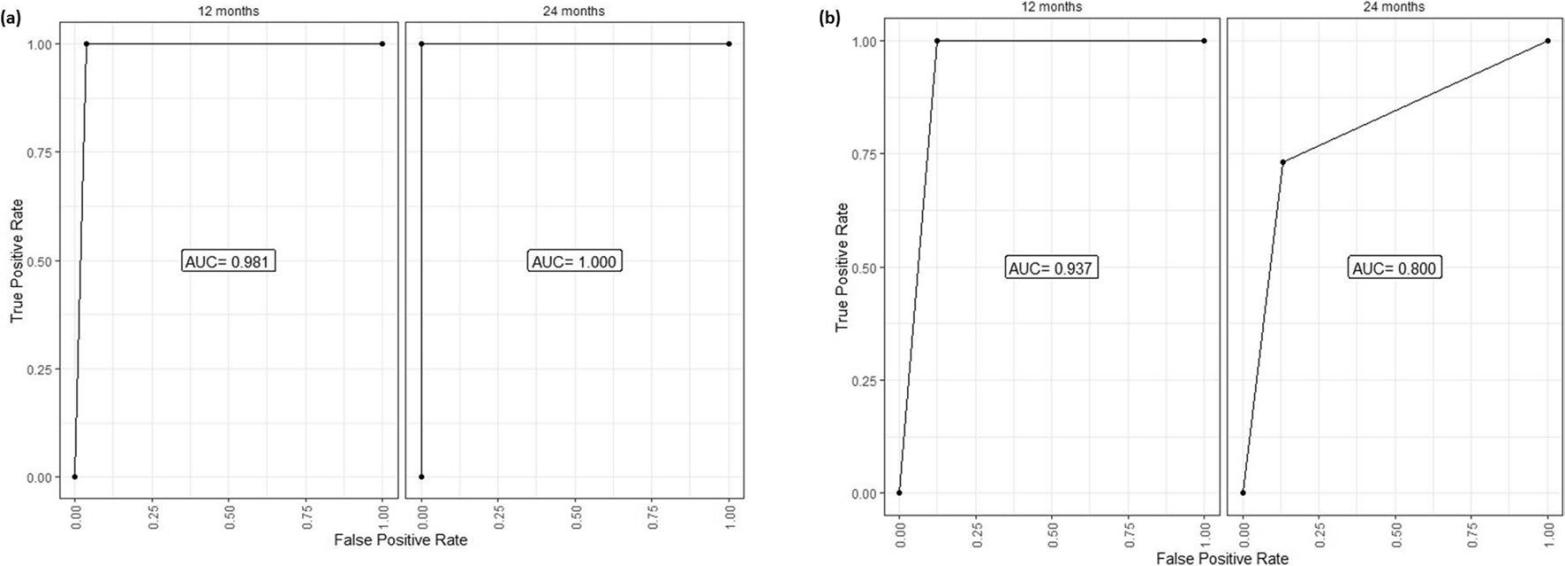
Time-dependent receiver operating curve (ROC) for prediction of overall survival based on **a** MYD88 genotype and **b** TP53 genotype

For predicting overall survival, the AUC values for MYD88 mutation demonstrated an excellent discriminative ability for predicting overall survival at both 12 and 24 months. The AUC for the 12-month ROC curve was 0.981. The AUC for the 24-month ROC curve was 1, which means that the model perfectly predicts the patients who die or survive within 24 months (Fig. 3a). For TP53, the AUC values were 0.937 and 0.800 at 12 and 24 months (Fig. 3b).

**Figure 4:**
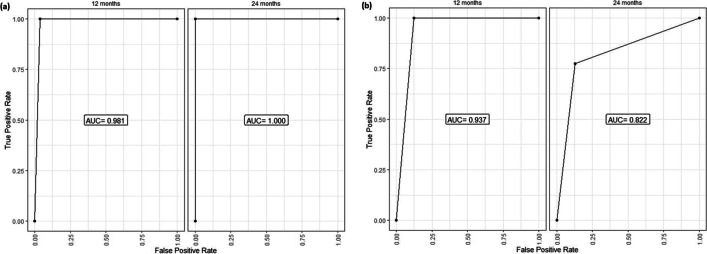
Time-dependent receiver operating curve (ROC) for prediction of progression-free survival based on **a** MYD88 genotype and **b** TP53 genotype

Likewise, there was a significantly lower 2-year progression-free survival in MYD88 mutant vs. wild cases (25% vs. 100%, *P*<0.001); hence, MYD88 genotype was associated with good discriminatory power for progression-free survival. The AUC estimates were 0.981 and 1.00 at 12 and 24 months, respectively, as plotted in Fig. 4a. Regarding TP53, there was a significantly lower 2-year progression-free survival in mutant TP53 vs. wild cases (62% vs. 97%, *P*<0.001), as the TP53 biomarker demonstrated a high predictive power for progression-free survival at both 12 and 24 months. The AUC values for P53 were 0.937 and 0.822 for 12 and 24 months, respectively as plotted in Fig. 4b.

When comparing mutant vs. wild cases, there was a significantly lower rate of 2-year survival (63% vs. 100%, *P*<0.001) and 2-year progression-free survival (57% vs. 100%, *P*<0.001). There were also significant differences in the 2-year survival (*P*<0.001) and 2-year progression-free survival (*P*<0.001) among the different patterns of mutations. The lowest 2-year survival and 2-year progression-free survival were presented in the MM group (43% and 29%, respectively). WW and TP53 mutant groups were presented with 100% 2-year survival and 2-year progression-free survival, as shown in Table 5.

**Table 5:** The 2-year overall survival and progression in different mutant cases

Characteristic	2-year survival		2-year progression-free survival	
	Survival probability	*P* value^1^	Survival probability	*P* value^1^
Overall	88% (79%, 99%)		86% (77%, 97%)	
MYD88		**<0.001**		**<0.001**
Wild	100% (100%, 100%)		100% (100%, 100%)	
Mutant	33% (12%, 96%)		25% (7.5%, 83%)	
TP53		**<0.001**		**<0.001**
Wild	96% (89%, 100%)		97% (90%, 100%)	
Mutant	70% (49%, 100%)		62% (40%, 95%)	
Genotype		**<0.001**		**<0.001**
Wild	100% (100%, 100%)		100% (100%, 100%)	
Mutant	63% (41%, 96%)		57% (35%, 90%)	
Pattern of mutation		**<0.001**		**<0.001**
WW	100% (100%, 100%)		100% (100%, 100%)	
MM	43% (18%, 100%)		29% (8.9%, 92%)	
MYD88 mutant	NA		NA	
P53 mutant	100% (100%, 100%)		100% (100%, 100%)	

^1^Log-rank test

The median survival for all the mutant cases (*N*=16) was 21 months. The median survival for wild cases (*N*=33) was not reached. The median survival for the MM subgroup (*N*=7) was 7 months, the mutant-MYD88-only group (*N*=2) was 10 months, and the mutant-TP53-only group (*N*=7) is 24 months.

**Figure 5:**
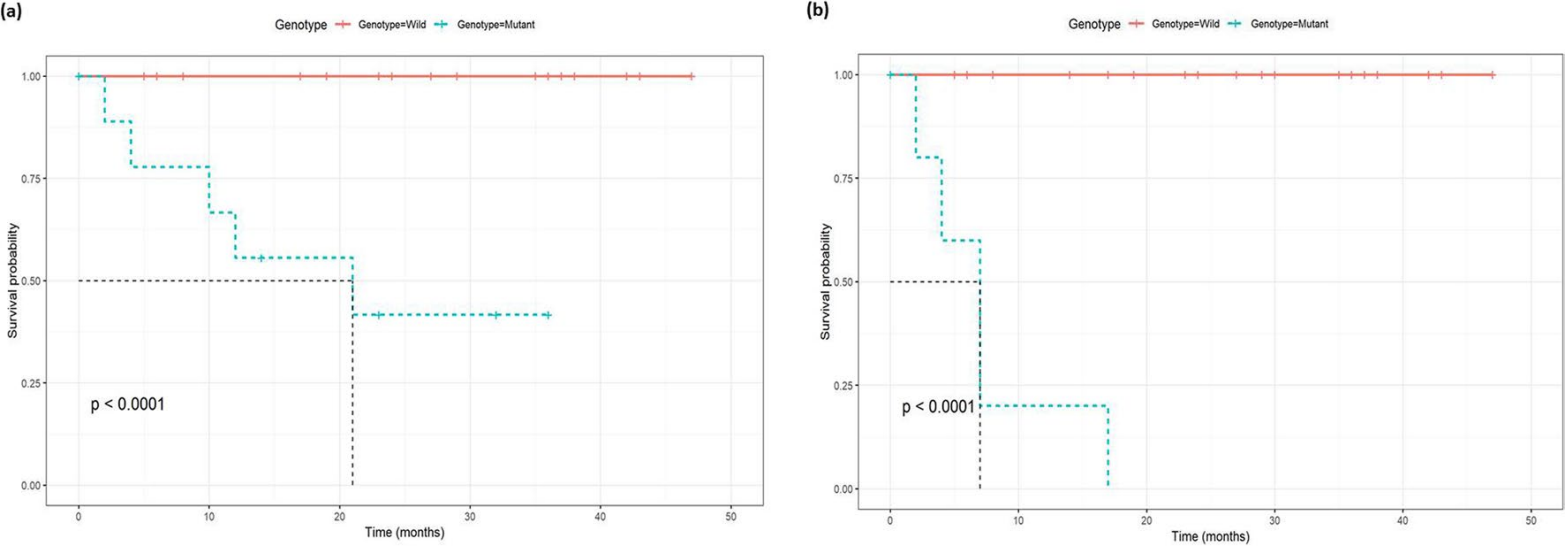
Kaplan-Meyer survival curves comparing **a** overall survival in wild vs. mutant genotypes, **b** progression-free survival in wild vs. mutant genotypes

**Figure 6:**
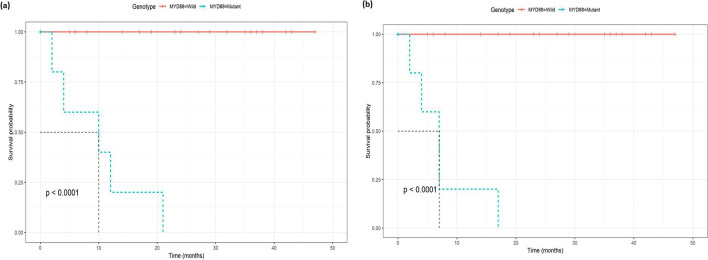
Kaplan-Meyer survival curves comparing **a** overall survival in MYD88 wild vs. MYD88 mutant genotypes and **b** progression-free survival in MYD88 wild vs. MYD88 mutant genotypes

**Figure 7:**
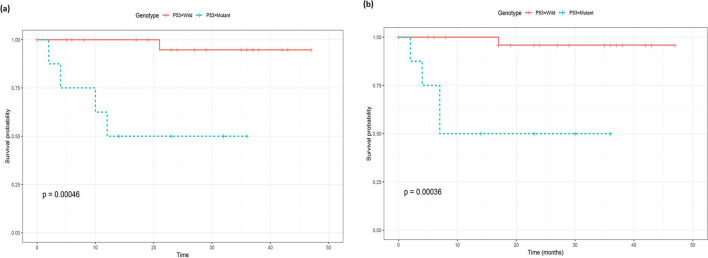
Kaplan-Meyer survival curves comparing **a** overall survival in TP53 wild vs. TP53 mutant genotypes and **b** progression-free survival in TP53 wild vs. TP53 mutant genotypes

There was a statistically lower overall survival (*P*<0.001) and progression-free survival (*P*<0.001) in mutant vs wild cases (Fig. 5a and b). Mutant MYD88 cases presented significantly lower overall survival (*P*<0.001) and progression-free survival (*P*<0.001) compared to wild cases (Fig. 6a and b). Likewise, mutant TP53 cases presented significantly lower overall survival (*P*<0.00046) and progression-free survival (*P*<0.00036) compared to wild cases (Fig. 7a and b).

The univariate COX regression identified TP53 mutations (HR: 17.6%, 95% CI, 2.15–144, *P*=0.008), MYD88 mutations (HR: 40, 95% CI, 4.86–329, *P*<0.001), and the total number of cycles received (HR: 0.63, 95% CI, 0.42–0.93, *P*=0.02) as the significant predictors of the time to mortality. Alternatively, the significant predictors of the time to progression were only TP53 mutations (HR: 12.9, 95% CI, 2.77–60.5, *P*=0.001), MYD88 mutations (HR: 31.7, 95% CI, 6.66–151, *P*<0.001), ECOG score 2–3 (HR: 3.93, 95% CI, 1.14–13.6, *P*= 0.031). The multivariate models demonstrated that MYD88 could predict the hazard of both death and progression alone (Model 1). There is no additional benefit when combining MYD88 with TP53 and/or IPI (models 2 and 3) in predicting the hazards of both death and progression (Table 6).

**Table 6:** Multivariate COX-proportional hazard predicting the overall survival

Characteristic	HR^1^	95% CI^1^	*P* value
MYD88 only			
MYD88 (Model 1)			
Wild	—	—	
Mutant	40.0	4.86, 329	**<0.001**
MYD88 + TP53 (Model 2)			
MYD88			
Wild	—	—	
Mutant	21.5	1.60, 290	**0.021**
TP53			
Wild	—	—	
Mutant	2.64	0.19, 36.7	0.5
MYD88 + P53 + IPI (Model 3)			
MYD88			
Wild	—	—	
Mutant	22.0	1.57, 308	**0.022**
TP53			
Wild	—	—	
Mutant	2.73	0.17, 44.3	0.5
IPI score^2^	0.97	0.45, 2.07	>0.9

^1^*HR*, hazard ratio, *CI*, confidence interval

^2^In order to allow for IPI incorporation into COX regression models, IPI scores were dichotomized into two categories: reference category (IPI 0–2) and second category (IPI 3–5)

## Discussion

The prognostic mutations and many precipitated genes in DLBCL have been recognized in recent years. The expression of their encoded proteins with a probable relationship to patient outcome is mainly unknown [19].

In the current study, MYD88 and TP53 mutations were identified in 18.73% and 28.57% of the patients, respectively. The estimated prevalence of both mutations through our study coincides with the current literature [12, 15, 20–22] (Xu et al., Chapugy et al., Nago et al., Oliver et al., and Leroy et al.).

It was previously found by Nago et al. [20] that mutations of MYD88 protein were in 19% of DLBCL patients. Other next-generation sequence studies by Xu et al. and Chapugy et al. have recently demonstrated similar frequencies of mutated MYD88 (15–18%) in large cohorts of DLBCL [12, 15]. These data were similar to current study findings with 18.73% mutated MYD88 in the DLBCL group.

The frequency of TP53 mutations among different studies found in DLBCL patients varies (from 5 to 23%) [21, 22]. In our samples, we could identify 28.6%. Such divergent percentages can be explained by the different techniques (like IHC and DNA sequencing) used to detect TP53 mutations and DLBCL genetic heterogeneity.

A study published by Mohamed and his colleagues showed that MYD88 genetic mutation was positively associated with higher ECOG scores; the high score (72.4%) had high mutation rates compared with the low score (27.6%), also associated with late stages and higher IPI score [19]. That was similar to our study that demonstrated a significant association of MYD88 positive mutations with poorer ECOG status, late stages, and high IPI score. Also, another analysis conducted by De Groen and his colleagues, involving a total of 275 DLBCL patients, showed that DLBCL patients with MYD88 mutation were significantly associated with high IPI score risk groups [23].

Multiple studies by Fernandez et al., Choi et al., and Kraan et al. demonstrated that patients with MYD88 mutation are older and have extranodal involvement and high serum LDH [24–26]. In contrast, the current data found no significant difference as regard age, LDH, and extranodal involvement between mutant and wild groups.

Similar to this study’s results, a study published by Zenz and his colleagues showed that TP53 mutation was associated with higher ECOG status and IPI-Scores [16]. However, Leroy et al. demonstrated that TP53 mutant and wild-type did not differ significantly in their main clinical characteristics [22].

The prognostic value of the MYD88 mutation and its correlation with OS has been a matter of debate, as some studies have reported that MYD88 mutation is significantly associated with low survival rates [24, 27, 28], while others identified no effect of MYD88 mutation on the survival of DLBCL patients [29, 30].

Multivariate analysis of the current study demonstrated that MYD88 mutations could predict the hazard of both death and progression alone and can be considered as an independent prognostic factor as the analysis showed that there is no additional benefit when combining MYD88 with TP53 and/or IPI (models 2 and 3) in predicting the hazards of both death or progression of disease with significant *P* value at 0.022 and 0.006, respectively. Similarly, a study published in 2020 by Vermaat and his colleagues showed that IPI (HR 1.77, 95% CI, 1.47–2.13) and the MYD88 mutation (HR 1.83, 95% CI, 1.19–2.80) were independent prognostic factors and had a similar impact on OS [31]. Additionally, another analysis by De Greon et al. showed that the performance of the IPI score is improved by adding MYD88 mutant as a poor risk factor [23].

This current study identified that patients with TP53 mutations included had 17.6 times more risk of death than patients without mutation (*P* value = 0.008), and that was similar to a study conducted by Kerbauy et al. that also identified an increased risk of death in patients with TP53 mutations [32].

The analysis of this study demonstrated that the mutant MYD88 was associated with worse 2-year PFS and OS rates versus wild type. The correlation of MYD88 mutations with an inferior overall survival is matched with several studies (De Greon et al., Vermaat et al., and Yu et al.) [10, 23, 31] On the other hand, a study by Xu et al. [12] found that no statistically significant correlation between MYD88 mutation and overall survival.

Due to this controversy and debate on the actual role of MYD88 mutation as a prognostic factor and its association with OS, more studies and investigations are required.

A prospective cohort of newly diagnosed DLBCL patients treated with RCHOP demonstrated that TP53 mutations are correlated with disease progression and poor survival and survival analysis for patients with wild-type TP53 had significantly better OS (*P* =0.0041) and PFS (*P* =0.0084) [33]. Also, several studies showed a significant impact of the TP53 mutations on OS of DLBCL patients [22, 34, 35] and that was similar to our results that showed worse 2-year PFS and OS rates regarding mutant TP53 with a significant *P* value less than 0.001. However, there were other studies different from our study results with insignificant difference for prognosis and survival [17, 36].

The study findings demonstrated a correlation between the presence of TP53 mutation and poor response rate to chemotherapy. This was matched with other studies by Cunningham et al. and Pfreundshuh et al. indicating TP53 mutations correlation with an unfavorable response to standard regimen RCHOP [37, 38].

Unexpectedly, the study results of Tamimi and his colleagues revealed a better overall survival rate for patients having TP53 mutations compared with patients’ wild-type TP53 gene [39]. So like MYD88 mutation, data concerning the survival and prognosis of TP53 mutation are conflicting and controversial.

The main limitations of this work include the data heterogeneity as we included all stages of DLBCL. Also, we used real-time PCR to detect gene expression; accordingly, we recommend performing a large prospective multi-centric study using next-generation sequencing to better detection of the gene expression that will help in future detection of DLBCL and prediction of survival.

Among the strengths of our study stands out the fact that this is the first study evaluating the significance of mutated MYD88 and TP53 in DLBCL patients in Egypt through extracting a liquid biopsy and measuring their sensitivity and specificity to correlate their prognostic impact. Also, a further study is in progress to compare used methods in the current work (HRM-PCR and restriction enzyme) to detect mutations in extracted tissue with paired cfDNA as to assure its sensitivity in both types of samples. In addition, our study shows that the incorporation of the mutational status of MYD88 and TP53 (especially MYD88) into a clinical/biochemical risk score as the IPI is feasible. They can be used in predicting disease progression and overall mortality, as our data demonstrated their significant sensitivity, specificity, and prognostic accuracy vs. IPI score. This encourages for more studies to test the utility of these gene mutations in diagnosis of DLBCL.

## Conclusion

Diffuse large B cell lymphoma is a highly heterogeneous lymphoid neoplasm with variations in gene expression profiles and genetic alterations.

Myeloid differentiation gene 88 and TP53 genes are common to be expressed and mutated in DLBCL patients that can affect prognosis and survival. Mutant MYD88 and TP53 showed their prognostic importance for worse survival outcomes, even next to other clinical prognostic factors like IPI. Study results provide a rationale for including MYD88 and TP53 mutational analysis (especially MYD88 mutations) in the routine sub-classification of DLBCL, to improve prognosis, as well as to guide future treatment strategies.

## Data Availability

The author agreed not to disclose their data.
